# Brain dynamics in ASD during movie‐watching show idiosyncratic functional integration and segregation

**DOI:** 10.1002/hbm.24009

**Published:** 2018-03-05

**Authors:** Thomas A.W. Bolton, Delphine Jochaut, Anne‐Lise Giraud, Dimitri Van De Ville

**Affiliations:** ^1^ Institute of Bioengineering École Polytechnique Fédérale de Lausanne (EPFL) Lausanne Switzerland; ^2^ Department of Radiology and Medical Informatics University of Geneva Geneva Switzerland; ^3^ Department of Neuroscience University of Geneva Geneva Switzerland

**Keywords:** autism spectrum disorders, dynamic functional connectivity, idiosyncrasy, inter‐subject functional correlation, sliding window, naturalistic movie, underconnectivity theory of autism

## Abstract

To refine our understanding of autism spectrum disorders (ASD), studies of the brain in dynamic, multimodal and ecological experimental settings are required. One way to achieve this is to compare the neural responses of ASD and typically developing (TD) individuals when viewing a naturalistic movie, but the temporal complexity of the stimulus hampers this task, and the presence of intrinsic functional connectivity (FC) may overshadow movie‐driven fluctuations. Here, we detected inter‐subject functional correlation (ISFC) transients to disentangle movie‐induced functional changes from underlying resting‐state activity while probing FC dynamically. When considering the number of significant ISFC excursions triggered by the movie across the brain, connections between remote functional modules were more heterogeneously engaged in the ASD population. Dynamically tracking the temporal profiles of those ISFC changes and tying them to specific movie subparts, this idiosyncrasy in ASD responses was then shown to involve functional integration and segregation mechanisms such as response inhibition, background suppression, or multisensory integration, while low‐level visual processing was spared. Through the application of a new framework for the study of dynamic experimental paradigms, our results reveal a temporally localized idiosyncrasy in ASD responses, specific to short‐lived episodes of long‐range functional interplays.

## 1 INTRODUCTION

Autism spectrum disorders (ASD) are characterized by a large set of neurodevelopmental brain alterations leading to socio‐communicative impairments and stereotypic behaviors (American Psychiatric Association, 2013). ASD affect 1 of 45 children according to the most recent estimates (Zablotsky, Black, Maenner, Schieve, & Blumberg, 2015), and remain mechanistically poorly understood. However, because a notable fraction of candidate genes relate to synaptic transmission (Persico & Napolioni, 2013; Krumm, O'Roak, Shendure, & Eichler, 2014), ASD are typically perceived as a system‐level condition, which gave rise to many functional magnetic resonance imaging (fMRI) studies attempting to decipher how the autistic brain works.

Accordingly, coordinated processing between separate brain regions, known as *functional connectivity* (FC), that is, typically quantified by correlational measures of statistical interdependency (Friston, 1994), has been assessed in resting‐state (Di Martino et al., 2014; Gotts et al., 2012; Supekar et al., 2013) and in a variety of tasks probing speech comprehension (Just, Cherkassky, Keller, & Minshew, 2004), visuomotor performance (Mizuno, Villalobos, Davies, Dahl, & Müller, 2006; Turner, Frost, Linsenbardt, McIlroy, & Müller, 2006; Villalobos, Mizuno, Dahl, Kemmotsu, & Müller, 2005), visuospatial abilities (Damarla et al., 2010; Liu, Cherkassky, Minshew, & Just, 2011), face processing (Kleinhans et al., 2008; Rudie et al., 2012), or executive functions (Just, Cherkassky, Keller, Kana, & Minshew, 2007; Kana, Keller, Minshew, & Just, 2007; Koshino et al., 2008). The big picture emerging from those reports is lowered FC between frontal and posterior brain regions (see Vissers, Cohen, & Geurts, 2012 for a review), as formulated in the *underconnectivity theory of autism* (Just, Keller, Malave, Kana, & Varma, 2012).

However, this view remains hotly debated, in light of the strong influence that methodological choices can have on obtained results (Jones et al., 2010; Müller et al., 2011; Nair et al., 2014). In particular, most task‐based studies focus their connectivity analyses on the regions that activate in response to the studied paradigm, ignoring functional interplays that may occur elsewhere in the brain (Wass, 2011). Also, despite the interest in task‐driven functional changes, the presence of intrinsic FC (iFC) variations (Damoiseaux et al., 2006; van den Heuvel & Pol, 2010; Hutchison, Womelsdorf, Gati, Everling, & Menon, 2013b) is never analytically accounted for; yet, iFC is altered throughout the brain in ASD (see for example Anderson et al., 2011; Cherkassky, Kana, Keller, & Just, 2006; Yahata et al., 2016). In some cases, ASD‐specific FC patterns put forward as caused by the studied task may thus reflect iFC group differences, or task‐driven effects may be overshadowed by iFC fluctuations and fail to be revealed.

On top of those limitations, traditional task‐based designs also selectively explore the functional underpinnings of one type of stimulus, although in a real life setting, the brain is constantly bombarded by a large array of sensory, social and emotional cues that may combine nonlinearly (Friston, Mechelli, Turner, & Price, 2000). Exploring how resulting FC patterns emerge at the whole‐brain level is an essential next step in our understanding of the autistic brain.

A well‐suited paradigm for this purpose is the viewing of a naturalistic movie (Campbell et al., 2015; Hasson, Malach, & Heeger, 2010). By correlating a given regional time course of activity across subjects, a measure of response homogeneity can be obtained. This approach revealed globally more idiosyncratic activity in ASD than in typically developing (TD) individuals (Hasson et al., 2009; Salmi et al., 2013). However, as a movie involves a continuously changing sequence of temporally overlapping cues, to which the brain dynamically adjusts, such stationary analyses (i.e., through one estimate for the whole task duration) may fall short of an accurate characterization.

Recently, Simony et al. (2016) extended this method into *inter‐subject functional correlation* (ISFC) to monitor the cross‐subject reliability of regional communication within default mode network regions during narrative comprehension. In this technique, FC is computed between the regional time courses sampled from distinct subjects. As a result, sources of FC that are temporally uncorrelated across individuals, such as iFC changes, are damped out, and only correlated effects, such as those elicited by a time‐locked paradigm, remain. Dynamic functional connectivity approaches (see Preti, Bolton, & Van De Ville, 2016 for a recent review) can then be applied, through consecutive measurements on progressively shifted temporal subwindows, to quantify the dynamics of ISFC throughout the course of the paradigm at hand.

Here, we computed ISFC in sliding window fashion on a movie‐watching dataset for which TD and ASD individuals were exposed to an audio–visual scientific documentary containing a rich array of visual, auditory, social and emotional cues, and devised a metric capturing dynamic brain responses to the movie as ISFC transients. We compared those responses across groups to examine how current views on FC alterations and response heterogeneity in autism would translate to this more complex dynamic setting.

## 2 MATERIALS AND METHODS

### 2.1 Subject information and data acquisition

Sixteen typically developing participants (TD) and 15 subjects diagnosed with primary autism disorder (ASD) with language impairments, as assessed by DSM‐IV criteria and confirmed with the Autism Diagnostic Interview‐Revised (ADI‐R; Lord, Rutter, & Couteur, 1994), participated to the study, which was approved by the local ethics committee (Biomedical Inserm protocol C08–39). All subjects, or legal representatives, provided their written informed consent.

In all but 3 subjects, IQ measures were acquired with a short form of the WAIS‐III scale (Weschler, 2000). In all participants except one TD and four ASD individuals, the Autism Spectrum Quotient (AQ; Baron‐Cohen, Wheelwright, Skinner, Martin, & Clubley, 2001) was also obtained. In all but three ASD subjects, scores from the ADI‐R were collected as well, spanning four different areas of impairment (verbal and nonverbal communication, social skills, repetitive and stereotyped behaviors).

For each subject, the data were acquired in three separate runs: the first (RUN_1_) and second (RUN_2_) were combined movie‐watching/resting‐state (RS) acquisitions, with the movie displayed from 5 to 353 s (348 s = 5.8 min duration), and the RS scanning lasting from 386 to 678 s (292 s = 4.9 min). The third run (RUN_3_) was a pure RS acquisition, which lasted for 310 s (5.2 min). All RS runs were conducted with eyes closed, instructing the subjects not to fall asleep. For all runs, subjects were also asked to refrain from moving as much as possible. RUN_3_ was not recorded in 4 subjects due to claustrophobia. Part of this data was already previously published in a combined EEG/fMRI analysis of speech processing (Jochaut et al., 2015).

Three hundred and forty echoplanar fMRI image volumes (Tim‐Trio; Siemens, 40 transverse slices, voxel size = 3 mm × 3 mm × 3 mm, repetition time = 2 s, echo time = 50 ms, field of view = 192) were acquired during RUN_1_ and RUN_2_, and 155 volumes during RUN_3_. A 7 min anatomical T1‐weighted magnetization‐prepared rapid acquisition gradient echo sequence (176 slices, voxel size = 1 mm × 1 mm × 1 mm, field of view = 256) was also acquired at the end of scanning.

**Figure 1:**
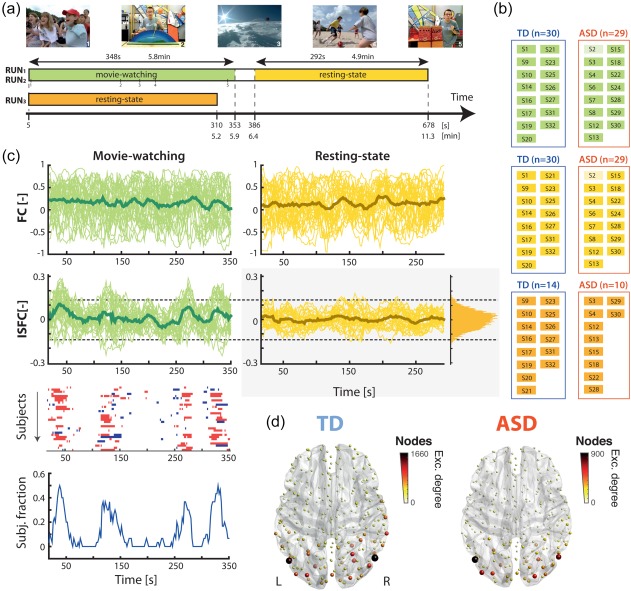
Recording paradigm and ISFC analysis. (a) Each subject underwent three separate acquisition runs: in the first two (RUN_1_, RUN_2_), 5.8 min of movie‐watching were followed by 4.9 min of eyes closed resting‐state. In the third one (RUN_3_), there was only a 310s‐long resting‐state acquisition. The movie watched by the subjects incorporated various types of stimuli, such as emotional scenes (example frame 1), scientific explanations (example frames 2 and 5), landscape depictions (example frame 3), or interactions between children and one of the presenters (example frame 4). (b) Separate ISFC computations were performed on the TD and ASD populations, for three settings: the movie‐watching sections (first half of RUN_1_ and RUN_2_, green), the resting‐state subparts following movie‐watching (second half of RUN_1_ and RUN_2_, yellow), and the purely resting‐state runs (RUN_3_, orange). The indices and amount of retained subjects for each setting are written down, and a more transparent box highlights cases where only one run (RUN_1_) was used. Blue and red rectangular contours mark the sets of runs selected for a given ISFC computation. (c) Example connectivity time courses for TD subjects (thin lines) and their average (thick line), computed with a standard sliding window methodology (top row) or with ISFC (bottom row), on the movie‐watching sections (left, green curves) or the subsequent resting‐state sections (right, yellow curves). To threshold a movie‐watching ISFC time course, a null distribution of values gathered from RS ISFC measurements (right‐hand side, light gray box) is used (dashed lines highlight example thresholds). This results, for each subject, in an ISFC time course with highlighted positive/negative excursions (+1/−1) that can be used either to count the number of excursions that occurred, or to track the fraction of subjects exhibiting significant ISFC changes at a given time point. (d) For the TD (left) and ASD (right) populations, average nodal degree for excursion number [Color figure can be viewed at http://wileyonlinelibrary.com]

The movie watched by the participants was an audio–visual scientific documentary for youngsters about the dangers of sun exposure, and was the same in RUN_1_ and RUN_2_. Participants were instructed to attentively watch the program, and were informed that they would have to briefly summarize its content after the MRI session. Two male presenters alternated in the movie, with the first providing oral explanations on scientific concepts, and for this purpose, often resorting to shape‐ and color‐filled panels. The second was recurrently interacting with a group of children through games and emotional chats. Landscape depictions, and narrations from a third female voice, completed the movie time. Example scenes from the documentary are presented in Figure 1a, along with a schematic depiction of the acquisition timings. It can be watched at https://miplab.epfl.ch/index.php/miplife/research/supplement-asd-study.

### 2.2 Preprocessing

Preprocessing of the data was performed separately for each run, using SPM12 (http://www.fil.ion.ucl.ac.uk) and a combination of in‐house MATLAB scripts and scripts from the DPARSFA toolbox (Yan & Zang, 2010). Functional volumes were realigned to the first image and co‐registered to the structural image, which was segmented to derive the nonlinear deformation field between native and MNI spaces. Voxel‐wise functional time courses were detrended, and constant, linear, quadratic, average cerebrospinal fluid (CSF) and average white matter (WM) time courses were regressed out. To generate CSF and WM time courses, we averaged the signal contained in conservative masks from the DPARSFA toolbox.

To ensure that only runs linked to minimal motion were retained for analysis, we quantified the percentage of frames with instantaneous framewise displacement (FD) larger than 0.5 mm (Power, Barnes, Snyder, Schlaggar, & Petersen, 2012), and excluded all runs with more than 30% of scrubbed frames. With this criterion, we kept 30 RUN_1_ (15 TD), 29 RUN_2_ (15 TD) and 24 RUN_3_ (14 TD) recordings. Age, IQ, AQ, and ADI‐R scores for all subjects are summarized in Table 1, along with mean FD (excluding scrubbed frames) for all the runs retained for analysis. Mean FD did not differ significantly between the TD and ASD groups (*p* = .08) as assessed by a group × run ANOVA design, and subjects from both groups were matched for age (*p* = .24, two‐tailed *t*‐test), gender (all males) and handedness (all right‐handed). Both populations significantly differed for IQ (*p* = 1.93 × 10^−4^), prompting us to apply additional steps to consider solely non IQ‐related connections in our analyses (see Section 2.5).

**Table 1:** Demographic, cognitive and motion‐related properties of the analyzed subjects

Subject information			Cognitive scores								Mean framewise displacement		
Index	Group	Age	IQ	ADIb	ADIcnv	ADIcv	ADId	ADIt_1_	ADIt_2_	AQ	RUN_1_	RUN_2_	RUN_3_
1	TD	20.17	106							19	0.11 (0.06)	0.08 (0.04)	n.a.
2	ASD	20.63	50	2	16	12	n.a.	7	35	n.a.	0.14 (0.08)	*0.19 (0.1)*	n.a.
3	ASD	27.17	90	3	24	11	17	8	43	n.a.	0.12 (0.06)	0.12 (0.07)	0.12 (0.06)
4	ASD	20.65	82	4	13	7	n.a.	6	26	n.a.	0.1 (0.06)	0.09 (0.07)	0.09 (0.05)
6	ASD	22.65	79	20	11	15	2	33	48	26	0.17 (0.08)	0.15 (0.07)	n.a.
7	ASD	27.78	n.a.	n.a.	n.a.	n.a.	n.a.	n.a.	n.a.	n.a.	0.25 (0.12)	0.28 (0.11)	n.a.
8	ASD	19.84	110	23	11	17	4	38	55	28	0.14 (0.09)	0.16 (0.1)	n.a.
9	TD	27.82	97							20	0.14 (0.07)	0.12 (0.06)	0.14 (0.05)
10	TD	18.95	97							13	0.14 (0.05)	0.15 (0.07)	0.16 (0.08)
12	ASD	17.64	66	28	11	17	5	44	61	28	0.19 (0.09)	0.17 (0.1)	0.16 (0.09)
13	ASD	15.62	80	17	5	12	8	30	42	21	0.17 (0.09)	0.15 (0.09)	0.11 (0.06)
14	TD	23.8	102							14	0.1 (0.05)	0.13 (0.08)	0.13 (0.07)
15	ASD	17.08	124	22	14	21	11	47	68	33	0.11 (0.07)	0.09 (0.05)	0.09 (0.04)
16	TD	16.09	114							12	0.14 (0.06)	0.16 (0.08)	0.17 (0.07)
17	TD	20.33	105							6	0.08 (0.04)	0.1 (0.06)	0.14 (0.08)
18	ASD	15.67	57	23	5	16	10	38	54	26	0.1 (0.05)	0.11 (0.05)	0.12 (0.06)
19	TD	17.69	112							12	0.07 (0.03)	0.07 (0.04)	0.07 (0.03)
20	TD	18.62	123							9	0.14 (0.07)	0.13 (0.07)	0.13 (0.06)
21	TD	17.63	92							11	0.11 (0.05)	0.11 (0.06)	0.1 (0.05)
22	ASD	16.24	85	20	14	23	3	37	60	43	0.16 (0.09)	0.17 (0.1)	0.14 (0.08)
23	TD	27.83	n.a.							n.a.	0.22 (0.12)	0.21 (0.12)	0.2 (0.11)
24	ASD	20.73	31	34	14	25	8	56	81	31	0.15 (0.1)	0.12 (0.09)	n.a.
25	TD	38.88	94							7	0.15 (0.07)	0.13 (0.05)	0.1 (0.05)
26	TD	17.9	n.a.							14	0.11 (0.04)	0.11 (0.05)	0.13 (0.08)
27	TD	27.83	97							14	0.13 (0.05)	0.14 (0.06)	0.13 (0.05)
28	ASD	17.46	120	25	17	22	2	44	66	21	0.14 (0.07)	0.1 (0.05)	0.11 (0.05)
29	ASD	40.47	75	36	14	21	7	57	78	32	0.14 (0.07)	0.14 (0.07)	0.13 (0.07)
30	ASD	17.58	91	25	14	18	5	44	62	27	0.12 (0.07)	0.11 (0.08)	0.11 (0.07)
31	TD	40.88	95							10	0.15 (0.06)	0.13 (0.05)	0.14 (0.05)
32	TD	16.91	127							9	0.11 (0.07)	0.1 (0.06)	0.08 (0.04)

*Note*. Abbreviations: IQ, intellectual quotient; ADIb, social component of the Autism Diagnostic Interview‐Revised; ADIcnv, nonverbal communication component of the Autism Diagnostic Interview‐Revised; ADIcv, verbal communication component of the Autism Diagnostic Interview‐Revised; ADId, repetitive and stereotyped behaviors component of the Autism Diagnostic Interview‐Revised; ADIt_1_, total Autism Diagnostic Interview‐Revised score without verbal component; ADIt_2_, total Autism Diagnostic Interview‐Revised score with verbal component; AQ, autism spectrum quotient; TD, typically developing; ASD, autistic.

The subjects did not differ in terms of age (*p* = .24) or mean framewise displacement (*p* = .08), but did in terms of IQ (*p* = 1.93 × 10^−4^). All participants were right‐handed males. Framewise displacement values are expressed in italics for the runs that were discarded for the analyses, and the values between parentheses indicate standard deviation.

In addition, we also conducted our analyses on a lower set of subjects, discarding the runs with more than 10% of scrubbed frames. This yielded a total of 15 RUN_1_ (7 TD), 16 RUN_2_ (8 TD) and 15 RUN_3_ (7 TD) recordings. In this setting as well, mean FD did not differ significantly between the groups (*p* = .27), which were matched for age (*p* = .48), gender and handedness. Results are presented in Supporting Information, Figure 1.

From the voxel‐wise data of each kept run, we performed regional averaging using a previously derived functional parcellation of the brain (*2‐level temporal correlation* algorithm from Craddock, James, Holtzheimer, Hu, & Mayberg, 2012, which we downloaded at https://www.nitrc.org/projects/cluster_roi). We used a 300‐region parcellation, of which *n*_ROI_ = 270 different regions of interest were present across all of the subjects that we analyzed. For each run, the MNI space atlas was inverse warped into native space using the deformation field acquired upon segmentation. Atlased time courses were scrubbed, discarding all frames with FD>0.5 mm (time *t*), and the following ones (time *t* + 1), and re‐estimating them through cubic spline interpolation. Finally, regional time courses were high‐pass filtered at 0.05 Hz.

To rearrange the indices of all atlas nodes in a topographically meaningful order, we computed the Euclidean distance between the centers of gravity of all region pairs, and constructed an adjacency matrix **A** according to $A_{ij}=e^{-\frac{d_{ij}^{2}}{\sigma^{2}}}$, where *d_ij_* is the Euclidean distance between regions *i* and *j*, and σ is the average distance across all region pairs. Nodes of the atlas were rearranged in ascending Fiedler vector values, as computed on the normalized graph Laplacian of the adjacency matrix (Von Luxburg, 2007), which resulted in an arrangement from occipital (low indices) to frontal (high indices) areas. We manually compiled a list of all regions with their index, MNI coordinates, structural brain location—as established through matching with the AAL atlas (Tzourio‐Mazoyer et al., 2002), and key functional roles—as extracted with the *neurosynth* platform (Yarkoni, Poldrack, Nichols, Van Essen, & Wager, 2011), which can be found in Supporting Information, Table 1.

### 2.3 Sliding window ISFC computations

To track FC changes upon movie‐watching, while at the same time minimizing the impact of dynamic fluctuations of intrinsic functional networks, we applied a sliding window inter‐subject functional correlation (ISFC) approach (Simony et al., 2016). For each region pair, connectivity was iteratively computed over a gradually shifted rectangular temporal window of length *W* = 20 s, the inverse of the lowest frequency still present in the data (Leonardi & Van De Ville, 2015), with a step size of 2 s. We used Pearson correlation coefficient as a measure of connectivity.

Instead of computing connectivity estimates within the same subject, which would amount to a classical sliding window paradigm (Allen et al., 2014), in ISFC, a regional time course is compared to another sampled from a distinct subject, and the final estimate is obtained as the average of the computed pairwise correlations. Formally, if $x_{i}^{\left[s\right]}(t)$ denotes the activity of region *i* for subject *s* at time *t*, an ISFC connectivity estimate between regions *i* and *j* for this subject is given by

$$ISFC_{ij}^{\left[s\right]}\left(\tilde{t}\right)=\;\frac{\sum_{k\in G}\rho \left(x_{i}^{\left[s\right]}\left(t:t+W\right),\;{\;x}_{j}^{\left[k\right]}\left(t:t+W\right)\right)}{N_{G}}.$$

Here, G denotes the reference group of subjects to which *s* is compared, *N*_G_ is the number of subjects in this group, and ρ(***x*_1_**,***x*_2_**) is the Pearson correlation coefficient computed between ***x*_1_** and ***x*_2_**. The full inter‐subject functional correlation matrix **ISFC** (size *n*_ROI_ × *n*_ROI_) is not symmetric, but for simplicity, we considered the symmetrical matrix given by ½ (**ISFC** + **ISFC** ^T^).

Due to the averaging process, intrinsic dynamic fluctuations, which are uncorrelated across subjects, will be damped. ISFC is specifically sensitive to time‐locked changes across subjects; in this case, we thus hope to capture connectivity changes driven by the movie, which was the only synchronized stimulus across runs.

ISFC was computed separately on TD and on ASD subjects because we reasoned that both populations may exhibit distinct patterns of connectivity changes, and we were interested in characterizing possible group differences in inter‐individual reliability of ISFC changes. We separately computed ISFC for TD and ASD subjects on (1) the movie‐watching subparts of RUN_1_ and RUN_2_ recordings, which are the ones considered in the presented analyses; (2) the RS subparts of the same runs, which are used for the selection of significant movie‐watching ISFC excursions (see Section 2.4); and (3) the purely RS RUN_3_ recordings, which are also used in the same thresholding process. The ISFC computational pipeline is summarized in Figure 1b.

In each of those cases, to ensure that ISFC estimates were not biased by the presence of a few outlier data points (Byrge, Dubois, Tyszka, Adolphs, & Kennedy, 2015), we used a bootstrapping approach where ISFC was computed 1,000 times, assessing synchrony with a different reference group at each fold. This reference group was randomly sampled from the full TD (for TD subjects) or ASD (for ASD subjects) population, using the recordings from 6 subjects (3 RUN_1_ and 3 RUN_2_). The results did not change when using more or less subjects (from 4 to 8).

### 2.4 Extraction of ISFC excursions

In Figure 1c, we illustrate the process of thresholding ISFC time courses to pinpoint significant excursions. For an indicative TD connection, ISFC time courses over the movie‐watching or RS time intervals (RUN_1_ and RUN_2_) are compared to FC estimates computed in within‐subject fashion, for which iFC fluctuations remain. In this latter case, both the movie‐watching and RS settings yield time courses with a positive average across runs, and a similar dispersion. This indicates the dominating influence of intrinsic dynamics over movie‐driven changes.

In the ISFC case, however, the RS time courses fluctuate around 0, and show lower fluctuations than their movie‐watching counterparts; this is because resting‐state dynamics and other uncorrelated effects across runs have been filtered out, enabling to more clearly track movie‐related connectivity fluctuations. Our aim was to determine, on the movie‐watching subparts of RUN_1_ and RUN_2_ recordings, when significant increases or decreases in ISFC occurred.

We hypothesized that different connections would show different levels of responsiveness to the movie, and so, assessed excursion significance separately for each connection. In each case, our null hypothesis was the absence of any movie‐driven ISFC change. To generate an appropriate null distribution, we aggregated the ISFC estimates from all available subjects from (1) the RS subparts of RUN_1_ and RUN_2_ and (2) the RUN_3_ data.

A Kolmogorov–Smirnov test revealed that in the majority of connection cases (26,544 of *n*_CON_ = 36,315), the null hypothesis of similar null distributions across groups could be rejected (*p* < .05). Thus, we resorted to separate null distributions instead of combining them. In total, this amounted to 6,184 (TD) and 5,462 (ASD) ISFC measurements per connection. Our significance thresholds were the 0.02^th^ and 99.98^th^ percentiles of the null distribution. We tagged the movie‐watching ISFC time courses with −1/1 if there was a significant negative/positive ISFC excursion, and with 0 if the null hypothesis could not be rejected (no significant excursion).

From this point, we computed the sum of significant ISFC excursions (notwithstanding their sign) in each subject for a summarizing measure of responsiveness to the movie (Section 3.1). To gain a more dynamic insight, we also determined the fraction of subjects showing a significant transient at each time point, a value that can range from −1 (all subjects show a significant ISFC *decrease*) to 1 (all subjects show a significant ISFC *increase*). We related individual connection time courses (Section 3.2) or whole‐brain spatial ISFC excursion patterns at key time points (Section 3.3) to specific movie stimuli.

### 2.5 Statistical assessment of excursion number group differences

To assess whether a given connection would differ across groups in terms of excursion number, we used a two‐way ANOVA design, with group (TD vs ASD) as first factor and run (RUN_1_ vs RUN_2_) as second factor. Bonferroni correction was applied for the *n*_CON_ tests performed in parallel.

Because our two groups were not matched for IQ, a fraction of group‐specific connections are likely to relate to IQ rather than to the ASD phenotype *per se*. To account for this and focus our analyses on non IQ‐related connections in our discussion, we correlated (Spearman rank correlation) the excursion numbers of each significant connection with (1) IQ scores within the TD group, (2) IQ scores within the ASD group, and (3) IQ scores of the whole population after having regressed out the IQ group difference. As soon as the connection under investigation showed a significant correlation (uncorrected *p* value of 0.05 or lower) for any of those 3 assessments, we deemed it IQ‐related and did not include it in our discussion. Group‐specific connections found to relate to IQ (a total of 623) are displayed in Supporting Information, Figure 2.

Furthermore, to ensure that our findings were not impacted by possible residual differences in motion across groups, we computed subject‐wise FD on the frames that were not scrubbed, and performed statistical assessments with or without this factor as a covariate. The presented results include this step, but the pool of found group‐specific connections was similar without (Supporting Information, Figure 3).

### 2.6 Analysis of excursion number time courses

When referring to specific moments of excursion number time courses, we always refer to the last frame of the movie that was included in the considered ISFC estimate. However, for our interpretation of what movie subpart occurred at that moment, we considered a hemodynamic delay of roughly 5 s (Friston et al., 2000), and took into account the 20 s temporal interval of the movie captured by the considered ISFC estimate. For example, a state expressed at 100 s relates to the movie interval going from 75 to 95 s.

### 2.7 Additional validation and clarification of the findings

A difference in ISFC excursions for a given connection may arise because of two separate phenomena: a lowered/absent response to the movie in one of the groups, or a more idiosyncratic response in this group. To disentangle those two factors, we repeated our analyses by computing ISFC (Section 2.3) using the same reference group of only TD subjects across both subject populations, following the example of Byrge et al. (2015). An absent or lowered response to the movie would translate into a group difference in this second setting, while a larger heterogeneity of the responses would not. Those results are presented in Supporting Information, Figures 6 and 7.

Furthermore, to verify that our results were not simply the result of ASD and TD subjects looking differently at the movie, and given the absence of eye‐tracking data for the analyzed populations, we selected the 7 nodes from the used atlas falling within the *Calcarine* AAL brain region (nodes 1, 2, 3, 6, 16, 36 and 40), devoted to low‐level visual processing, and computed inter‐subject correlation for each node within the TD and the ASD groups (using the same bootstrapping scheme as before, and a reference group made of only TD subjects). Of the 7 cases, only node 1 resulted in a significant group difference (*p* = .0133), prompting us to believe that the way subjects watched the movie only mildly affected our results.

## 3 RESULTS

### 3.1 Group differences in ISFC excursions

To study the changes in ISFC driven by the movie, we determined the time points showing significant ISFC excursions for each subject, and counted how many such events occurred (see Sections 2.3 and 2.4). For both groups, the nodes implicated in the most responsive connections (Figure 1d) were located in occipital or temporal brain areas involved in visual processing (nodes 20, 27, 29, 42 and 43). The TD group also displayed some auditory processing foci (e.g., node 95).

**Figure 2:**
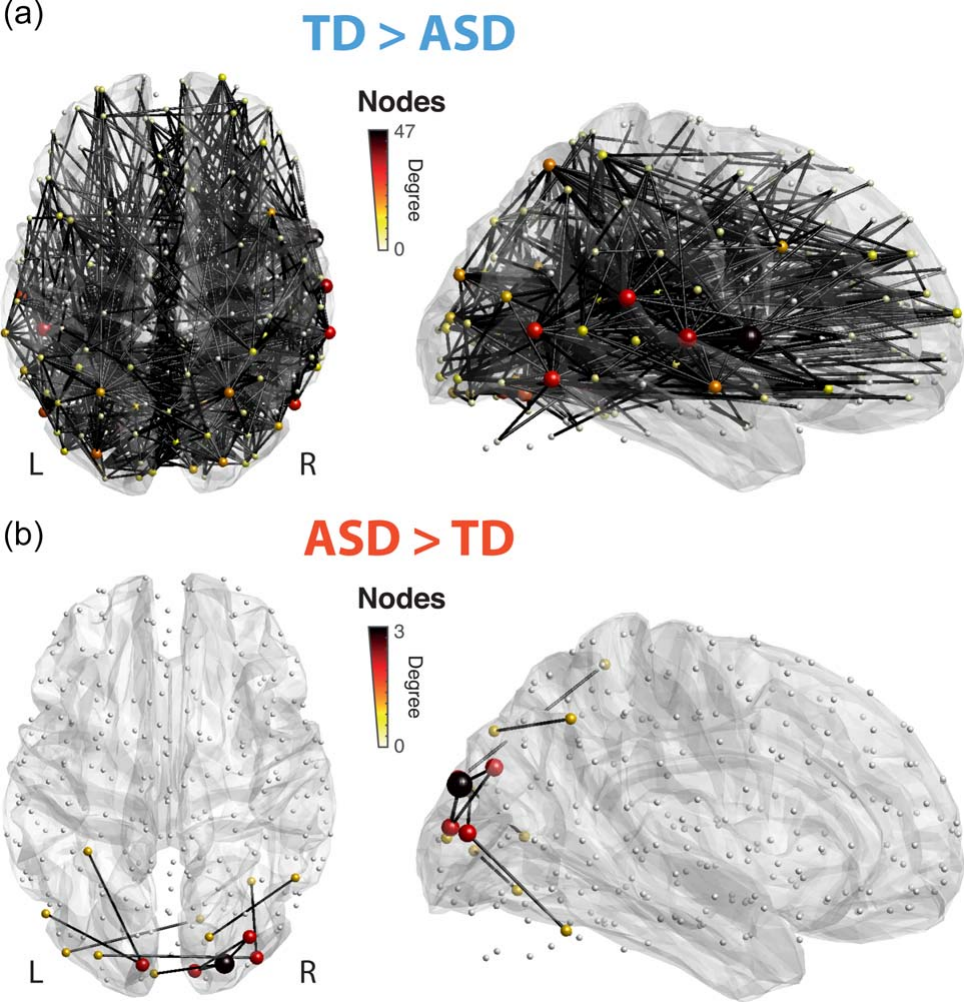
Responsiveness to the movie. Connections showing significantly more ISFC excursions in the TD group (a) or the ASD group (b) displayed on a horizontal (left) or sagittal (right) brain view. The size and color of the nodes are proportional to the number of incoming significant connections. The right side is facing towards the reader in sagittal depictions [Color figure can be viewed at http://wileyonlinelibrary.com]

Overall, 707 connections (1.95% of the total assessed amount) differed in excursion number across groups without being related to IQ (see Section 2.5). The large majority of them (697) showed more ISFC excursions in TD than in ASD subjects. The most implicated regions for TD > ASD connections (Figure 2a) were part of the superior temporal (nodes 95, 98, 131 and 168), middle temporal (nodes 42, 43, 69, 72, 76, 102 and 147), or inferior temporal (nodes 45 and 48) cortices, but there were also lingual (nodes 14, 18, 19 and 56), occipital (nodes 8 and 15) and calcarine (node 6) contributions. All lengths of connections could be found, but there was a larger amount of long‐range links, resulting in an elevated average of 82.9 ± 34.5 mm.

The few connections (10) showing more ISFC excursions in ASD individuals (Figure 2b) all spanned the occipital brain, with the majority of them involving superior occipital (node 8), middle occipital (nodes 13, 20 and 21), inferior occipital (node 24), fusiform (node 27), middle temporal (node 43) or inferior temporal (node 45) areas devoted to visual processing. The right precuneus (node 44) and the left superior parietal cortex (node 91) were also among the implicated regions. ASD‐specific connections were of significantly shorter range than TD ones (41.9 ± 17.4 mm, *p* = 3.91 × 10^−4^ upon two‐tailed *t*‐testing). The occipital localization of connections showing more excursions in ASD—as compared to more broadly spread ones for TD subjects—was also observed on a restricted dataset with more stringent motion criteria (Supporting Information, Figure 1).

When a similar reference group made of only TD subjects was used for ISFC computations instead (see Section 2.7), group differences became more balanced (Supporting Information, Figure 6): 50 connections showed significantly more ISFC excursions in TD individuals, primarily involving occipital (nodes 29, 32 and 42) and temporal (nodes 95, 98, 131 and 168) areas. Seventy‐six connections showed a larger amount of excursions in ASD subjects, and mostly related to lingual (nodes 18 and 19), middle occipital (nodes 13, 15 and 21), superior occipital (node 8) and calcarine (node 1) subparts from the visual cortex. In addition, some parietal (nodes 58 and 59) and temporal (nodes 72 and 127) foci were also seen.

### 3.2 Temporal profile of ISFC excursion time courses

**Figure 3:**
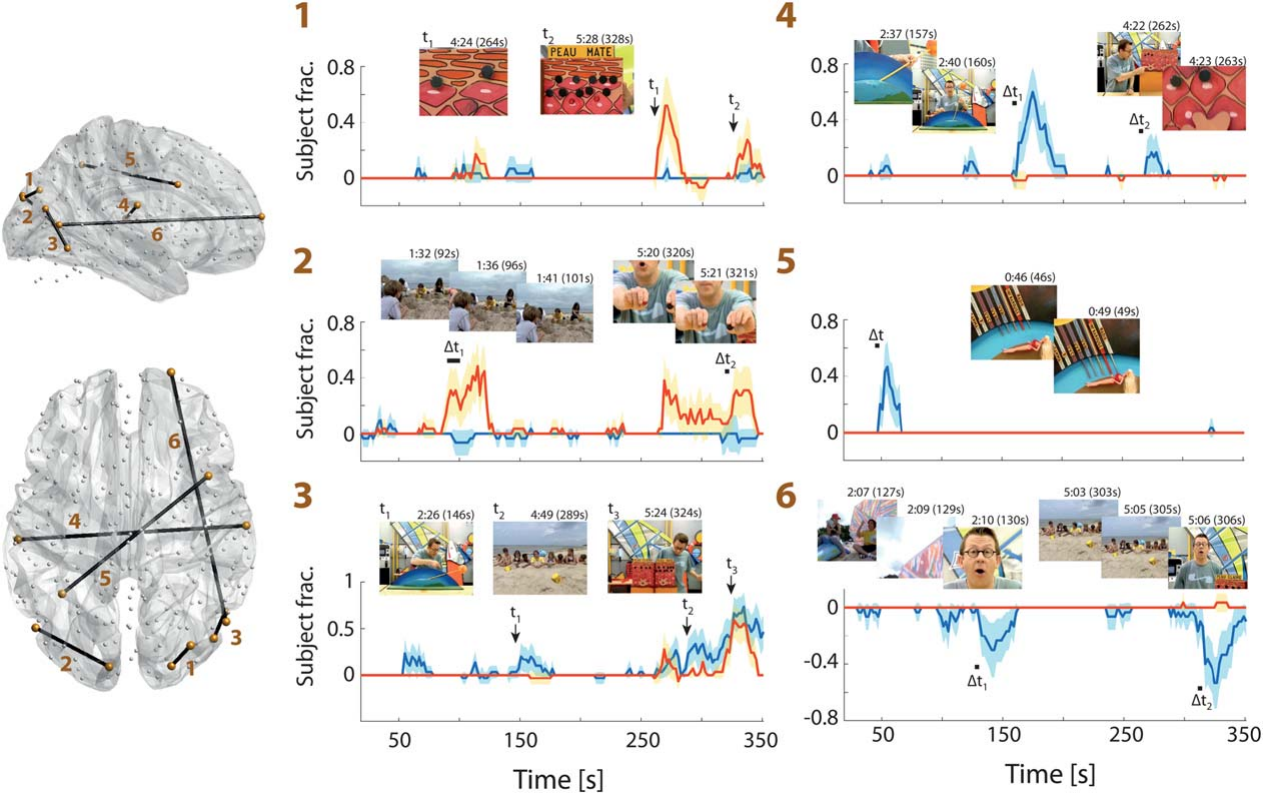
ISFC excursion time courses for selected connections. (Left) Sagittal and horizontal brain slices depicting the connections (from 1 to 6) chosen for display. (Right) For TD (blue) or ASD (red) subjects, ISFC excursion time courses for the selected connections are displayed with two‐tailed 95% confidence intervals. In each case, for the most salient ISFC transients, the movie time points that triggered the changes are highlighted by an arrow (for one frame) or a horizontal bar (for a subset of frames). Illustrations of the related frames are also displayed at the top of each plot, along with their time of occurrence. Note that an ISFC estimate is drawn at the last movie time point that was included to compute it [Color figure can be viewed at http://wileyonlinelibrary.com]

To determine whether the ISFC excursions examined above were temporally locked across subjects, and to see whether they could be related to specific movie stimuli, we computed the fraction of subjects showing a significant transient at each time point. Indicative examples are displayed in Figure 3, and an exhaustive display of all connection time courses is shown in Supporting Information, Figure 5.

Connection 1 linked the right superior occipital cortex (node 8), implicated in motion perception (Klaver et al., 2008), to the right middle occipital cortex (node 21), an area involved in object detection (Burgund, Lugar, Miezin, & Petersen, 2003). Connection 2 involved the left cuneus (node 7), previously linked to peripheral vision (Stenbacka & Vanni, 2007), and a region of the left middle temporal cortex (node 43) implicated in motion processing (Kawawaki, Shibata, Goda, Doya, & Kawato, 2006). Both connections were part of the ones more responsive to the movie in ASD subjects, and showed clear temporal locking, with up to half of ASD individuals exhibiting a positive ISFC transient at the same time point. For connection 1, the largest peaks followed the presentation of geometrical shapes during the movie (at 264 and 328 s), while for connection 2, positive excursions were triggered after movie subparts during which moving stimuli were present in the background (92–101 and 320–321 s).

Connection 3 was significantly more responsive in TD subjects, and linked two regions from the visual system: one (node 29) related to visual context (Villarreal, Fridman, & Leiguarda, 2012), and one (node 48) involved in object recognition (Gruber, Trujillo‐Barreto, Giabbiconi, Valdés‐Sosa, & Müller, 2006). It underwent positive ISFC excursions following moments of the movie when objects could be perceived within a particular context (at 146, 289 and 324 s). TD subjects showed a response to all three stimuli, while ASD ones only reacted to the last one, which included geometrical shapes on a colorful display panel.

Connection 4 involved a region from the left auditory cortex (node 127), and a right post central area located in the somatosensory cortex (node 135). As such, it reflects FC across distinct sensory systems. In TD subjects, two significant moments of positive ISFC excursions followed particular instants from the movie (157–160 and 262–263 s) when one of the presenters touched an item, while at the same time or just afterwards, a novel sound cue accompanied the motion of the object.

Connections 5 and 6 are examples of interactions between sensory and frontal brain regions, showing positive or negative ISFC transients, respectively. They were only responsive in TD individuals. Connection 5 involved an inferior parietal region (node 73) related to the expectation of moving objects (Shulman et al., 1999), and a right frontal opercular area (node 196) linked to response inhibition (Sebastian et al., 2013). It peaked after one particular scene of the movie when colored lines were extending towards a doll, and abruptly stopped just in front (46–49 s). Connection 6 was between area V_5_ (node 42) and a right superior frontal region (node 266), and showed lowering of ISFC at times of the movie just after there was a sudden transition from a scene to a close‐up on one of the presenters (127–130 and 303–306 s).

### 3.3 Whole‐brain ISFC excursion patterns at key time points

**Figure 4:**
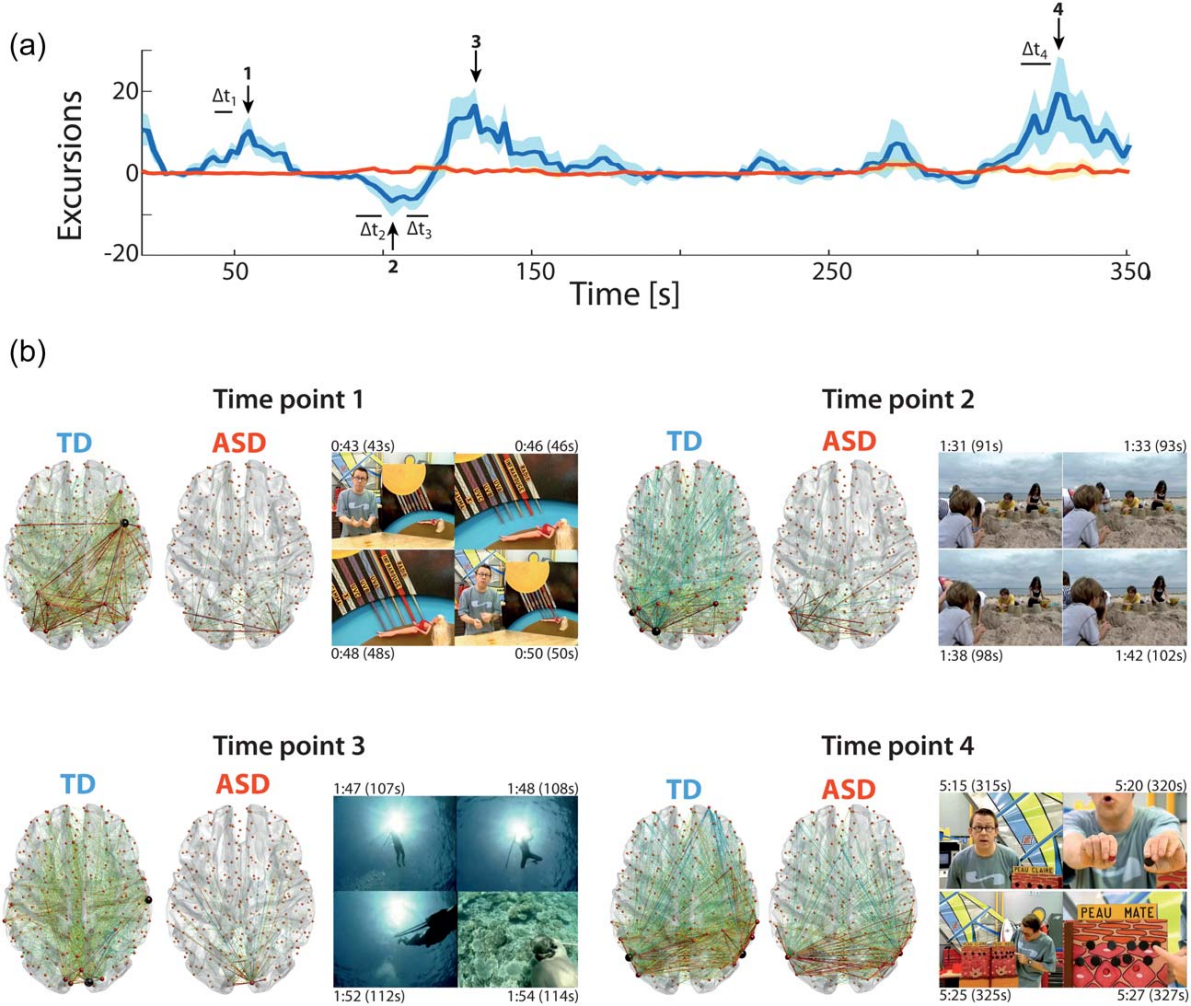
Whole‐brain ISFC transient patterns. (a) ISFC excursion time courses for TD (blue) or ASD (red) subjects, summed across all connections retained as group‐specific. Peak time points (from 1 to 4) are labeled by vertical arrows, and the movie frames that triggered the ISFC changes are labeled by horizontal bars. Note that an ISFC estimate is drawn at the last movie time point that was included to compute it. (b) For the selected time points, whole‐brain horizontal depiction of the connections that were undergoing significant ISFC excursions in TD (left slice) and ASD (right slice) subjects, and example frames from the movie time span that triggered the appearance of the pattern (right panel), with annotated times at which the frames occurred. Stroke thickness in the brain plots is proportional to the fraction of subjects that showed a significant ISFC increase (in red) or decrease (in blue). The size and color of the spheres are proportional to nodal degree. TD and ASD brain plots from the same time point are at the same scale, and can thus directly be compared for edge thickness and node size [Color figure can be viewed at http://wileyonlinelibrary.com]

To study whole‐brain ISFC excursion patterns at particularly salient time points, we summed the excursion time courses of all group‐specific connections (Figure 4a). Unsurprisingly given the prominence of TD > ASD connections, the resulting time course exclusively highlighted moments of whole‐brain ISFC changes occurring in TD individuals. More specifically, there was a broad increase in ISFC at three time points (labeled 1, 3 and 4), and one moment of widespread ISFC decrease (2).

Time point 1 (Figure 4b) followed the same movie subpart (43–50 s) as described above for connection 5. In TD subjects, it featured a rise in ISFC between occipital (nodes 15 and 18) and parietal (nodes 58, 59 and 73) regions implicated in the processing of moving stimuli, as well as with frontal areas involved in response inhibition (nodes 196 and 236). In ASD individuals, the rise stayed confined to purely occipital, low‐level visual regions (nodes 13, 20 and 43), strictly capturing the motion component of the stimulus.

Time point 2 relates to connection 2 described above (following the 91–102 s movie interval). In TD subjects, the occipito‐parietal visual circuitry showed enhanced ISFC (nodes 15, 20, 43 and 58), and at the same time, a decrease in synchrony with the frontal brain (nodes 236, 246, 260 and 264). Those long‐range effects were not observed in ASD subjects, for whom only increased ISFC of the visual circuitry (nodes 5, 8, 15, 20 and 43) was observed.

Time point 3 occurred following the only moment of the movie (107–114 s) when a female voice could be heard, while depictions of a moving diver in water were shown at the same time (notice the particularly bright stimulus caused by the sun reflecting in water). In TD subjects, there was a very strong ISFC increase in primary visual areas (nodes 1, 3, 19 and 27), and at the same time, in the auditory cortex (node 131). In the ASD case, only milder visual ISFC rises could be seen (nodes 3, 14, 16, 18 and 19).

Time point 4 occurred just after scientific explanations had been provided by one of the two presenters using geometrical shapes and a colorful panel (315–327 s). In both TD and ASD subjects, ISFC increases were seen within the visual circuitry (nodes 20, 42, 43, 48 and 69 for TD subjects; 20, 24, 29, 42, 43, 48 and 69 in ASD subjects), and in TD individuals, it was complemented by lowered ISFC with frontal regions (nodes 196 and 266).

When performing the above whole‐brain assessment on the same pool of 707 connections, but quantifying ISFC evolution with respect to a common reference group of healthy subjects (see Section 2.7), similar temporal profiles were observed across groups, with all four moments of whole‐brain ISFC changes present in comparable magnitude across populations (Supporting Information, Figure 7a). Further, ISFC patterns were also very similar, with both groups exhibiting qualitatively similar responses at the different key time points (Supporting Information, Figure 7b).

## 4 DISCUSSION

### 4.1 Revealing FC alterations in ASD during movie‐watching

Through ISFC, movie‐induced changes in connectivity can be located in time, and disentangled from overshadowing iFC fluctuations, so that the extent with which FC between different brain regions is influenced by the incoming stimulus can be accurately quantified. Harvesting this dynamic approach, we assessed whether there would be remnants of the global functional underconnectivity posited in ASD from stationary task‐based studies, and explored the possible idiosyncrasy of ASD responses in this dynamic setting.

Of the connections that were differently responding to the movie across groups, the large majority was significantly less recruited in ASD than in healthy individuals (Figure 2). The strong involvement of occipital and temporal brain regions, respectively related to the visual and auditory processing functions underlying movie‐watching, suggests that those may suffer alterations in ASD. Further, the overall elevated length of the involved connections implies that the deficit may reside in longer range communication across functional modules in the brain.

This globally stronger responsiveness of TD subjects, which was obtained by tracking ISFC excursions separately in ASD and TD populations, did not extend to a computational case involving a common reference group of healthy subjects (Supporting Information, Figure 6), where group differences were less numerous and more balanced. This hints at the exact nature of observed group differences: rather than originating from a difference in the average behavior of the groups, they result from a larger heterogeneity of responses within the ASD population than within the TD one.

Those points could be deepened by harvesting the dynamic potential of our ISFC excursion metric: at the level of individual connections, we observed TD‐specific increases in ISFC for a connection linking the somatosensory and auditory functional systems (Figure 3, connection 4), denoting their robust interplay following times of the movie when tactile and auditory cues jointly occurred. We also observed, solely in TD subjects, increases in ISFC upon response inhibition (connection 5), which can be regarded as another example of long‐range functional communication between sensory and frontal areas.

Importantly, those findings were not limited to a specific subset of connections, but involved the whole brain, as they were retrieved moving from a temporal snapshot perspective (tracking ISFC excursions at the level of one connection) to a spatial snapshot one, considering whole‐brain ISFC transient patterns at salient time points. In doing so, we observed that in several occasions, whereas connectivity within a given sensory system was similar in both TD and ASD subjects, the latter failed at appropriately engaging in ISFC changes with remote brain locations. In a situation where response inhibition was clearly witnessed in healthy individuals through enhanced ISFC between visual and frontal opercular regions (Figure 4, time point 1), ASD ones showed no such rise. At another moment when salient auditory and visual cues were provided together (time point 3), TD subjects showed prominent communication across sensory modules, while it did not happen in the ASD case. This last observation is well in line with recent findings of atypical multisensory integration in ASD (Stevenson et al., 2014). Importantly, as those interplays across functional systems were present in both groups of subjects, with similar dynamics, in the setting of a similar reference group of healthy subjects (Supporting Information, Figure 7), one should interpret the absence of response as a greater heterogeneity in the way ASD subjects responded to the movie.

Interestingly, this lower homogeneity in the recruitment of long‐range regional interactions in the ASD population did not only involve increases in ISFC: when background motion in the visual scene had to be suppressed, TD individuals exhibited prominent lowering of ISFC between the visual system and the rest of the brain, somehow secluding this network, but it was not the case in ASD subjects. This is consistent with previous work pointing at the fact that on top of functional integration, functional segregation is also impaired in ASD (Rudie et al., 2012).

In summary, because the dynamic responses seen in TD subjects were also retrieved in the ASD population upon the use of a similar reference group for ISFC computations (Supporting Information, Figure 7), our results do not lend support to the underconnectivity theory of autism (Just et al., 2012), tested here for the first time in the more realistic and dynamic movie‐watching setting. However, they show that the idiosyncrasy in brain responses previously reported across ASD subjects in stationary computational settings (Hasson et al., 2009; Hahamy, Behrmann, & Malach, 2015; Salmi et al., 2013) concerns precisely timed movie events involving functional integration and segregation.

On top of possible structural brain deficits, there is always the possibility that other factors external to the ASD phenotype itself contributed to the observed group differences. In our case, this is a particular concern as there was a significant difference in IQ between our studied TD and ASD populations. To deal with this issue as conservatively as possible, we considered all the connections that showed a group difference for our excursion number metric, and discarded any for which, at an uncorrected statistical level, there was any relationship with within‐group or whole‐population IQ. This ruled out almost a half of the connections found significant, and gives us confidence that the findings discussed here really relate to ASD. Incidentally, this is also seen in the type of reported alterations, which highlight sensory‐related processing rather than executive tasks.

Apart from the globally higher responsiveness seen in TD subjects, there was also a small subset of short‐range occipito‐occipital connections showing more ISFC excursions in ASD individuals (Figure 2b), extending along both the ventral and dorsal visual processing streams. From the analyzed excursion time courses (Figure 3), more vivid responses to geometrical shapes (connection 1) or to background motion in a visual scene (connection 2) were found among those upscaled functions in ASD. At the whole‐brain level (Figure 4), although cross‐network communication was consistently altered in ASD, the circuitry within the visual system appeared relatively spared.

Although not part of the core ASD symptoms, enhanced low‐level visual processing abilities are widely acknowledged (see Dakin & Frith, 2005 for a review), and translate into increased occipital activation upon various types of visual stimulation (Samson, Mottron, Soulieres, & Zeffiro, 2012), of which stronger occipital ISFC transients as seen here could be a consequence. Interestingly, it has recently been suggested that increased visual responsiveness to low‐level features, as reported in this study, may arise due to a larger noise in the primary visual system (Simmons et al., 2009; Lawson, Rees, & Friston, 2014). Along those lines, a larger intra‐individual variability of the evoked visual response has been reported in ASD (Dinstein et al., 2012; Haigh, Heeger, Dinstein, Minshew, & Behrmann, 2015).

### 4.2 Advantages and limitations of the methodology

Deploying our sliding window ISFC pipeline, we could specifically isolate the FC changes driven by a correlated source across all considered subjects (in our case, the movie). In this, the technique stands out in its ability to filter out uncorrelated sources of fMRI signal changes across subjects. One such source is intrinsic FC, which also shows complex dynamics (Hutchison et al., 2013a; Preti et al., 2016). On top of this, head motion is also increasingly understood to lead to severe artefacts in fMRI data if not properly accounted for (Power et al., 2012; Van Dijk, Sabuncu, & Buckner, 2012). While we considered motion‐matched subject populations, and despite scrubbing our analyzed data, the damping of possibly remaining motion‐related artefacts in the fMRI signals is another advantage of the ISFC approach, as different subjects show temporally uncorrelated motion patterns.

The price to pay for those advantages is a more complex interpretation to give to the results: as ISFC is computed through an averaging process across pairs of subjects, its changes must be understood as increases or decreases in synchrony with respect to the group. A consequence is that the conclusions drawn from an ISFC analysis relate to shared responses to the movie across the whole population of subjects on which ISFC is computed. As such, we may thus have been blind to some subject‐specific responses. In addition, our use of a bootstrapping approach in assessing ISFC actually ensures a limited impact of outlier subjects on the results. To further improve in this regard, the application of subsampling strategies to larger datasets could be considered in future work.

A limitation of the ISFC approach, shared by all sliding window schemes, is its reliance on second‐order rather than first‐order estimates, thus lowering the temporal resolution of the analyses, which is somewhat limiting in the context of a dynamic stimulus. In particular, this prevents the possibility to pinpoint one particular frame of the movie as driving a given ISFC configuration; rather, any of the data points contributing to the particular estimate may be involved, although we observed that in most occasions, the last time points to enter a given estimate were the most influential in driving ISFC transients.

To limit the impact of this lowered temporal resolution, while at the same time keeping enough data points for robust ISFC estimates, we opted here for a relatively short sliding window of 20 s. A possible extension to this work could be to probe how movie‐driven FC changes vary with frequency, for example by investigating whether there are also more slow‐paced, significant fluctuations induced in other brain systems as the ones described here. The use of time–frequency analysis strategies (Chang & Glover, 2010; Yaesoubi, Allen, Miller, & Calhoun, 2015) could fit this purpose, to the cost of a more complex set of data to analyze.

Another essential point pertaining to any sliding window analysis is the extent to which observed FC fluctuations are truly the reflection of dynamic changes in brain connectivity, rather than artefactual correlates of the employed methodology (Hindriks et al., 2016). In the resting‐state literature, several approaches have been suggested for this purpose, from the computation of FC across separate subjects (Keilholz, Magnuson, Pan, Willis, & Thompson, 2013) to the generation of appropriate null data with disrupted dynamics (Betzel, Fukushima, He, Zuo, & Sporns, 2016; Leonardi et al., 2013). Here, to extract significant ISFC excursions, we combined two approaches: the high‐pass filtering of preprocessed regional time courses with the inverse of the window length (set here to 20 s), which greatly limits the impact of spurious FC fluctuations (Leonardi & Van De Ville, 2015; Zalesky & Breakspear, 2015), and the use, as null data to threshold the movie‐watching ISFC time courses under the null hypothesis of no movie‐driven FC changes, of resting‐state segments from the same subjects, with identical acquisition parameters.

One possible caveat at this stage may lie in the fact that some of the resting‐state data (RUN_1_ and RUN_2_ recordings) were acquired immediately after watching the movie, making it possible that some movie‐related processing was still going on at the time. To try and compensate for this as much as possible, we also included RUN_3_ recordings, which were acquired separately from the movie‐watching runs, in the generation of connection‐specific null distributions. Also, while it is true that some movie‐relevant activity may have been missed, our null model would then be too conservative, rather than too permissive, and so the presented findings would still hold.

Finally, as a first analysis on our sliding window ISFC data, we computed excursion numbers on thresholded ISFC time courses, following the aforementioned significance assessment process. This metric can thus be viewed as providing information about the changes, or innovations, in the ISFC signal. In using this approach, we aimed at translating to the task‐based setting an emerging trend from the resting‐state fMRI field, where transients in connectivity (Betzel et al., 2016) or in activity (Karahanoğlu & Van De Ville, 2015) are being increasingly studied.

Two limitations from this measure, however, are its inability to disentangle ISFC increases from decreases, and the lack of exact timing information about the excursion time points. This is why we subsequently probed the dynamics of excursion time courses. In doing so, we observed that negative ISFC transients were part of the features distinguishing the TD and ASD groups, as they were more prevalent in the former. We could also successfully understand what types of stimulus in the movie would elicit a given ISFC change, both for specific connections or when probing whole‐brain responses.

Finally, another methodological direction that could be applied, in future work, to sliding window ISFC data is its disentanglement into a subset of temporally segregated whole‐brain connectivity states, as commonly employed in the resting‐state literature (see, e.g., Allen et al., 2014; Damaraju et al., 2014). By this mean, quantification and group comparison of the occurrence, timing and spatial characteristics of particular movie‐driven ISFC configurations could be achieved. We believe that the combined use of those different analytical strategies to probe other realistic paradigms opens up promising perspectives for understanding the dynamics of autism and other brain disorders.

## Supporting information

Additional Supporting Information may be found online in the supporting information tab for this article.

Supporting Information Figure 1

Supporting Information Figure 2

Supporting Information Figure 3

Supporting Information Figure 4

Supporting Information Figure 5

Supporting Information Figure 6

Supporting Information Figure 7

Supporting Information Table 1
