# Supplementary material for: Brain dynamics in ASD during movie‐watching show idiosyncratic functional integration and segregation
Source: Hum Brain Mapp. 2018 Mar 5;39(6):2391–404. doi: 10.1002/hbm.24009 (PMC5969252; doi:10.1002/hbm.24009)
Supplement: Supplementary file 8 — Supporting Information Table 1 [file HBM-39-2391-s008.docx]

| **Index** | **MNI coord.** | **Location (AAL atlas)** | **Function (*Neurosynth*)** |
| --- | --- | --- | --- |
| 1 | (-5, -93, 7) | L Calcarine | v1, primary visual, experimental conditions |
| 2 | (15, -95, -3) | R Calcarine | occipito temporal, category, occipito |
| 3 | (11, -92, 10) | R Calcarine | visual, visual cortices, early visual |
| 4 | (-11, -95, -8) | L Cuneus | disgust, interact, occipital |
| 5 | (-24, -95, 7) | L Occipital Mid | open, visual, dot |
| 6 | (1, -85, -13) | L Calcarine | mental imagery, names, younger adults |
| 7 | (-10, -89, 25) | L Cuneus | cuneus, sighted, suppressed |
| 8 | (22, -89, 23) | R Occipital Sup | vision, mt, visual |
| 9 | (31, -90, -8) | R Occipital Inf | inferior occipital, occipital, visual |
| 10 | (20, -85, -16) | R Lingual | disgust, engagement, videos |
| 11 | (-26, -91, -13) | L Lingual | face ffa, fusiform face, ffa |
| 12 | (4, -81, 24) | R Cuneus | highlighted, sighted, anterior prefrontal |
| 13 | (35, -87, 9) | R Occipital Mid | occipital, occipital cortex, allocation |
| 14 | (-7, -77, 5) | L Lingual | visual, visual cortices, cuneus |
| 15 | (-28, -86, 21) | L Occipital Mid | visual, occipital, vision |
| 16 | (10, -74, 8) | R Calcarine | visual, v1, early visual |
| 17 | (14, -81, -30) | R Cerebellum Crus 1 | episodic, performances, differs |
| 18 | (-18, -76, -12) | L Lingual | hemifield, lingual, visual cortex |
| 19 | (13, -71, -9) | R Lingual | lingual, executed, ba 10 |
| 20 | (-40, -85, 3) | L Occipital Mid | occipital, fusiform, visual |
| 21 | (32, -78, 29) | R Occipital Mid | shape, visual, object |
| 22 | (16, -79, 40) | R Cuneus | posterior parietal, parieto occipital, cortex ppc |
| 23 | (-12, -78, 39) | L Cuneus | parieto, parieto occipital, navigation |
| 24 | (43, -76, -10) | R Occipital Inf | ffa, face, fusiform |
| 25 | (35, -78, -28) | R Cerebellum Crus 1 | crus, cortico, cerebellar |
| 26 | (0, -68, -18) | Vermis 6 | cerebellum, movements, movement |
| 27 | (27, -69, -13) | R Fusiform | visual cortex, lingual, visual |
| 28 | (-38, -78, -14) | L Fusiform | fusiform, face, visual |
| 29 | (44, -75, 17) | R Occipital Mid | scenes, plays role, temporoparietal |
| 30 | (-33, -79, 34) | L Occipital Mid | episodic, episodic memory, memory |
| 31 | (-12, -67, 23) | L Cuneus | autobiographical memory, retrieval, episodic |
| 32 | (-43, -77, 21) | L Occipital Mid | sparse, intentions, angular gyrus |
| 33 | (1, -69, 37) | L Precuneus | retrieval, precuneus, autobiographical |
| 34 | (15, -65, 26) | R Precuneus | retrieval, recollection, memory |
| 35 | (22, -68, -29) | R Cerebellum 6 | cerebellum, crus, vi |
| 36 | (-16, -63, 4) | L Calcarine | eye movements, gaze, primary sensory |
| 37 | (-26, -71, 42) | L Parietal Inf | superior parietal, intraparietal, intraparietal sulcus |
| 38 | (1, -60, -4) | Vermis 4 5 | list, cerebellum, finger tapping |
| 39 | (31, -69, 43) | R Occipital Sup | parietal, superior parietal, anterior prefrontal |
| 40 | (20, -59, 8) | R Calcarine | maturation, resources, lingual |
| 41 | (44, -69, 35) | R Angular | default mode, default, independent component |
| 42 | (51, -66, 6) | R Temporal Mid | motion, mt, v5 |
| 43 | (-48, -69, 8) | L Temporal Mid | motion, mt, v5 |
| 44 | (10, -67, 52) | R Precuneus | parietal, parietal cortex, working memory |
| 45 | (-47, -66, -8) | L Temporal Inf | object, visual, objects |
| 46 | (-8, -66, 54) | L Precuneus | parietal network, parietal, location |
| 47 | (-26, -58, -12) | L Fusiform | fusiform, objects, selectivity |
| 48 | (50, -61, -8) | R Temporal Inf | visual, objects, occipito |
| 49 | (46, -61, -26) | R Cerebellum Crus 1 | crus, lobules, expertise |
| 50 | (33, -57, -21) | R Cerebellum 6 | cerebellum, fusiform, cerebellar |
| 51 | (-13, -56, -22) | L Cerebellum 4 5 | cerebellum, finger, vi |
| 52 | (1, -53, 17) | R Precuneus | default, default mode, mode |
| 53 | (52, -61, 24) | R Angular | default, mode, default mode |
| 54 | (-43, -66, 42) | L Angular | angular, default network, angular gyrus |
| 55 | (-47, -62, 22) | L Temporal Mid | semantic, junction, mentalizing |
| 56 | (24, -52, -10) | R Lingual | temporal lobes, parahippocampal, discriminate |
| 57 | (16, -52, -23) | R Cerebellum 4 5 | cerebellum, finger, motor |
| 58 | (25, -62, 57) | R Parietal Sup | eye movements, intraparietal, superior parietal |
| 59 | (-26, -61, 56) | L Parietal Sup | parietal, intraparietal, intraparietal sulcus |
| 60 | (8, -53, 37) | R Precuneus | mentalizing, beliefs, precuneus |
| 61 | (-7, -52, 36) | L Precuneus | mind, theory mind, mental states |
| 62 | (-12, -49, -1) | L Lingual | navigation, anterior hippocampus, connectivity posterior |
| 63 | (2, -49, -16) | Vermis 4 5 | vermis, cerebellum, lobules |
| 64 | (13, -47, 1) | R Lingual | retrosplenial, episodic, parahippocampal cortex |
| 65 | (37, -56, 49) | R Angular | parietal, intraparietal, task |
| 66 | (-48, -55, -19) | L Fusiform | word form, visual word, word |
| 67 | (-57, -56, -5) | L Temporal Inf | posterior temporal, meaning, semantic |
| 68 | (50, -55, 43) | R Parietal Inf | inferior parietal, parietal, frontoparietal |
| 69 | (60, -51, 6) | R Temporal Mid | temporal sulcus, sulcus psts, sulcus |
| 70 | (-54, -55, 32) | L Angular | default mode, default, mode |
| 71 | (8, -54, 64) | R Precuneus | navigation, switch, group healthy |
| 72 | (-56, -53, 12) | L Temporal Mid | sentence, temporal sulcus, sts |
| 73 | (-34, -52, 45) | L Parietal Inf | intraparietal, parietal, parietal cortex |
| 74 | (47, -47, -18) | R Temporal Inf | fusiform, face, fusiform gyrus |
| 75 | (-17, -45, -18) | L Cerebellum 4 5 | lobules, vi, crus |
| 76 | (51, -47, 16) | R Temporal Mid | junction, tpj, temporoparietal junction |
| 77 | (-7, -49, 52) | L Precuneus | separated, navigation, parietal lobules |
| 78 | (61, -48, -8) | R Temporal Mid | gestures, ipl, hippocampus |
| 79 | (-14, -52, 66) | L Precuneus | somatosensory, somatosensory cortices, pointing |
| 80 | (58, -48, 30) | R Supramarginal | stop signal, violations, signal task |
| 81 | (31, -42, -19) | R Fusiform | fusiform, anterior hippocampus, parahippocampal |
| 82 | (-38, -45, -19) | L Fusiform | fusiform, face, fusiform gyrus |
| 83 | (20, -48, 67) | R Parietal Sup | resonance compare, somatosensory, superior parietal |
| 84 | (6, -44, 52) | R Precuneus | precuneus, aberrant, abilities |
| 85 | (-50, -48, 47) | L Parietal Inf | parietal, parietal cortex, inferior parietal |
| 86 | (-3, -39, -27) | L Cerebellum 3 | vi, brainstem, cerebellum |
| 87 | (34, -45, 60) | R Parietal Sup | movements, hand, action observation |
| 88 | (-26, -38, -8) | L Parahippocampal | parahippocampal, medial temporal, parahippocampal cortex |
| 89 | (1, -38, 31) | L Cingulum Post | posterior cingulate, mode network, default mode |
| 90 | (27, -36, -5) | R Hippocampus | anterior hippocampus, hippocampus, hippocampal |
| 91 | (-32, -45, 60) | L Parietal Sup | intraparietal, premotor, hand |
| 92 | (39, -39, 44) | R Parietal Inf | intraparietal sulcus, intraparietal, parietal |
| 93 | (-58, -42, 30) | L Supramarginal | recording, supplementary, supplementary motor |
| 94 | (-60, -40, -9) | L Temporal Mid | memories, retrieval, parahippocampal cortex |
| 95 | (64, -38, 16) | R Temporal Sup | planum temporale, temporale, planum |
| 96 | (56, -35, 3) | R Temporal Mid | temporal sulcus, superior temporal, posterior superior |
| 97 | (54, -38, 45) | R Supramarginal | parietal, inferior parietal, inferior |
| 98 | (-48, -36, 18) | L Temporal Sup | auditory, planum, planum temporale |
| 99 | (-10, -32, -11) | L Cerebellum 4 5 | parahippocampal gyrus, midbrain, periaqueductal |
| 100 | (10, -39, 69) | R Postcentral | foot, sensations, limb |
| 101 | (13, -32, -11) | R Lingual | lobe mtl, mtl, medial temporal |
| 102 | (-64, -38, 7) | L Temporal Mid | superior temporal, auditory, speech |
| 103 | (17, -31, -25) | R Cerebellum 3 | navigation, correction, cerebellum |
| 104 | (-14, -32, 3) | L Thalamus | thalamus, binding, nucleus |
| 105 | (63, -33, -10) | R Temporal Mid | interpret, networks, intended |
| 106 | (-41, -36, 48) | L Postcentral | parietal, intraparietal, intraparietal sulcus |
| 107 | (-9, -33, 43) | L Cingulum Mid | healthy handed, self referential, referential |
| 108 | (-51, -33, 1) | L Temporal Mid | sentences, language, sentence |
| 109 | (-9, -36, 70) | L Paracentral Lobule | foot, limb, motor cortex |
| 110 | (15, -29, 5) | R Thalamus | thalamus, comprised, taste |
| 111 | (10, -31, 44) | R Cingulum Mid | sulcus psts, posterior cingulate, psts |
| 112 | (33, -28, -18) | R Parahippocampal | hippocampal, hippocampus, parahippocampal |
| 113 | (50, -29, 18) | R Temporal Sup | secondary somatosensory, somatosensory, somatosensory cortices |
| 114 | (41, -31, 56) | R Postcentral | dorsal premotor, contralateral, motor |
| 115 | (-33, -28, -18) | L Fusiform | hippocampal, hippocampus, formation |
| 116 | (26, -30, 65) | R Postcentral | primary motor, somatosensory, motor premotor |
| 117 | (61, -28, 30) | R Supramarginal | somatosensory, pain, imagine |
| 118 | (-25, -30, 65) | L Postcentral | primary motor, motor cortex, primary |
| 119 | (-54, -29, 41) | L Parietal Inf | hand, movements, motor |
| 120 | (-1, -23, -17) | - | midbrain, periaqueductal, brainstem |
| 121 | (51, -23, -4) | R Temporal Sup | superior temporal, temporal sulcus, speech |
| 122 | (-38, -25, 14) | L Heschl | auditory, listening, sound |
| 123 | (60, -21, -18) | R Temporal Mid | default, mode network, predictions |
| 124 | (-59, -26, 26) | L Supramarginal | somatosensory, pain, somatosensory cortex |
| 125 | (0, -25, 58) | L Paracentral Lobule | foot, sensorimotor cortex, motor imagery |
| 126 | (-39, -26, 57) | L Postcentral | motor, hand, finger |
| 127 | (-57, -24, 11) | L Temporal Sup | auditory, auditory cortex, sounds |
| 128 | (55, -23, 44) | R Postcentral | sensorimotor, parietal, younger adults |
| 129 | (38, -21, 13) | R Heschl | pain, sensation, painful |
| 130 | (-59, -23, -7) | L Temporal Mid | mind tom, tom, theory mind |
| 131 | (63, -20, 4) | R Temporal Sup | auditory, auditory cortex, sounds |
| 132 | (1, -20, -3) | - | midbrain, periaqueductal, striatum |
| 133 | (-10, -20, 10) | L Thalamus | thalamus, thalamic, finger |
| 134 | (1, -21, 33) | R Cingulum Mid | cingulate, signal task, stop signal |
| 135 | (61, -16, 19) | R Postcentral | somatosensory, tactile, somatosensory cortex |
| 136 | (6, -19, 71) | R Supp Motor Area | foot, limb, coordination |
| 137 | (-22, -16, -18) | L Hippocampus | hippocampus, hippocampal, episodic |
| 138 | (40, -17, 59) | R Precentral | ipsilateral, m1, motor |
| 139 | (16, -15, -19) | R Parahippocampal | amygdala, cortex amygdala, accumbens |
| 140 | (10, -16, 10) | R Thalamus | thalamus, thalamic, cortex thalamus |
| 141 | (-58, -16, -20) | L Temporal Mid | network dmn, default, dmn |
| 142 | (44, -16, 46) | R Precentral | cortical motor, motor task, motor cortex |
| 143 | (-47, -17, 44) | L Postcentral | motor, sensorimotor cortex, sensorimotor |
| 144 | (29, -14, -15) | R Hippocampus | hippocampus, hippocampal, medial temporal |
| 145 | (-42, -16, -1) | L Temporal Sup | posterior insula, auditory stimuli, block |
| 146 | (43, -13, -2) | R Temporal Sup | posterior insula, insula, brainstem |
| 147 | (59, -11, -11) | R Temporal Mid | listening, auditory, listened |
| 148 | (-9, -16, 71) | L Paracentral Lobule | primary motor, foot, motor cortex |
| 149 | (-6, -15, 44) | L Cingulum Mid | task difficulty, motor, posterior insula |
| 150 | (57, -9, -25) | R Temporal Mid | theory mind, mind, distress |
| 151 | (-26, -13, 65) | L Precentral | movement, dorsal premotor, m1 |
| 152 | (24, -12, 67) | R Frontal Sup | motor, premotor, primary motor |
| 153 | (55, -9, 37) | R Postcentral | primary somatosensory, somatosensory, motor |
| 154 | (-30, -11, -1) | L Putamen | putamen, basal, basal ganglia |
| 155 | (-59, -10, 13) | L Rolandic Oper | somatosensory, somatosensory cortex, speech production |
| 156 | (-41, -11, 13) | L Insula | somatosensory, insula, posterior insula |
| 157 | (8, -11, 45) | R Cingulum Mid | nervous, cingulate, undergoing |
| 158 | (-57, -9, 31) | L Postcentral | sparse, sensorimotor, somatosensory |
| 159 | (43, -8, 13) | R Insula | posterior insula, insular, insula |
| 160 | (-41, -9, 53) | L Precentral | motor, movements, movement |
| 161 | (-4, -10, 11) | LThalamus | thalamus, nucleus, insula anterior |
| 162 | (-56, -6, -10) | L Temporal Sup | spoken, comprehension, sentences |
| 163 | (30, -6, 2) | R Putamen | putamen, basal ganglia, ganglia |
| 164 | (-1, -6, -12) | - | hypothalamus, sexual, amygdala |
| 165 | (0, -6, 59) | L Supp Motor Area | motor, movements, supplementary |
| 166 | (61, -1, 22) | R Postcentral | speech production, production, speech |
| 167 | (30, -2, -23) | R Amygdala | amygdala, neutral, emotional |
| 168 | (58, -1, 4) | R Temporal Sup | heschl, pitch, speech production |
| 169 | (29, -3, 55) | R Frontal Mid | frontal eye, eye fields, eye |
| 170 | (-27, -4, 54) | L Frontal Mid | frontal eye, eye, parietal |
| 171 | (45, -1, 50) | R Precentral | eye movements, eye, eye fields |
| 172 | (-40, -1, -12) | L Temporal Sup | insula, pain, posterior insula |
| 173 | (-16, -2, -13) | L Amygdala | amygdala, emotional, emotion |
| 174 | (-29, 0, -23) | L Amygdala | amygdala, neutral, angry |
| 175 | (41, 1, -11) | R Insula | pain, insula, intensity |
| 176 | (55, 4, -14) | R Temporal Sup | superior temporal, acoustic, temporal sulcus |
| 177 | (17, 0, -12) | R Amygdala | amygdala, emotional, skin conductance |
| 178 | (-49, 1, 41) | L Precentral | premotor, premotor cortex, motor |
| 179 | (-9, 1, 68) | L Supp Motor Area | preparation, supplementary motor, premotor |
| 180 | (-54, 2, 4) | L Rolandic Oper | premotor, operculum, electrical |
| 181 | (1, -1, 0) | - | gamma, algorithm, mm |
| 182 | (13, 2, 66) | R Supp Motor Area | supplementary, supplementary motor, motor |
| 183 | (13, -1, 16) | R Caudate | caudate, caudate nucleus, nucleus |
| 184 | (-1, 0, 34) | L Cingulum Mid | cingulate, pain, middle cingulate |
| 185 | (54, 6, 34) | R Precentral | premotor, motor, movements |
| 186 | (-26, 2, 5) | L Putamen | putamen, basal ganglia, ganglia |
| 187 | (40, 6, 6) | R Insula | insula, pain, heat |
| 188 | (-38, 7, 53) | L Frontal Mid | reappraisal, tasks, eye fields |
| 189 | (-54, 7, 20) | L Precentral | phonological, premotor, inferior frontal |
| 190 | (-13, 3, 16) | L Caudate | caudate, striatal, caudate nucleus |
| 191 | (-38, 6, 6) | L Insula | insula, pain, painful |
| 192 | (-3, 5, 48) | L Supp Motor Area | motor, supplementary motor, supplementary |
| 193 | (-23, 9, 60) | L Frontal Mid | working memory, working, memory wm |
| 194 | (-24, 7, -5) | L Putamen | putamen, striatum, reward |
| 195 | (-42, 8, 31) | L Precentral | pain, cingulate, painful |
| 196 | (41, 9, 32) | R Frontal Inf Oper | interference, task, ifg |
| 197 | (25, 8, -1) | R Putamen | putamen, cortical subcortical, subcortical |
| 198 | (42, 15, -24) | R Temporal Pole Sup | pole, temporal pole, receive |
| 199 | (26, 11, 57) | R Frontal Sup | calculation, working, working memory |
| 200 | (53, 13, 15) | R Frontal Inf Oper | inferior frontal, inferior, handed |
| 201 | (39, 12, 51) | R Frontal Mid | postcentral, wm task, parietal |
| 200 | (53, 13, 15) | R Frontal Inf Oper | inferior frontal, inferior, handed |
| 203 | (50, 15, -3) | R Insula | insula, frontal operculum, anterior insula |
| 204 | (27, 15, -17) | R Insula | cortex insula, orbitofrontal, insula |
| 205 | (8, 11, 42) | R Cingulum Mid | supplementary, anterior cingulate, task |
| 206 | (-7, 14, 62) | L Supp Motor Area | remembering, autobiographical memory, autobiographical |
| 207 | (-28, 15, -16) | L Insula | orbitofrontal, orbitofrontal cortex, amygdala |
| 208 | (-7, 14, -8) | L Caudate | striatum, accumbens, nucleus accumbens |
| 209 | (10, 18, 61) | R Supp Motor Area | orbitofrontal cortex, response inhibition, orbitofrontal |
| 210 | (-41, 19, 41) | L Frontal Mid | autobiographical, prefrontal, bilinguals |
| 211 | (14, 14, 9) | R Caudate | caudate, nucleus, caudate nucleus |
| 212 | (11, 16, -5) | R Caudate | monetary, reward, striatum |
| 213 | (-13, 15, 4) | L Caudate | caudate, striatum, caudate nucleus |
| 214 | (-50, 22, 8) | L Frontal Inf Tri | inferior frontal, semantic, comprehension |
| 215 | (-27, 21, 50) | L Frontal Mid | word pairs, network connectivity, connectivity patterns |
| 216 | (42, 23, 39) | R Frontal Mid | frontoparietal, control network, item |
| 217 | (38, 24, -11) | R Frontal Inf Orb | anterior insula, ventrolateral, insula |
| 218 | (-39, 23, -12) | L Frontal Inf Orb | inferior frontal, semantic, dorsomedial prefrontal |
| 219 | (36, 22, 4) | R Insula | anterior insula, insula, insular |
| 220 | (-47, 23, 26) | L Frontal Inf Tri | demands, memory, words |
| 221 | (49, 25, 24) | R Frontal Inf Tri | distributed, inferior frontal, word |
| 222 | (-2, 18, 30) | L Cingulum Ant | cingulate, pain, anterior cingulate |
| 223 | (-34, 22, 3) | L Insula | anterior insula, insula, insula anterior |
| 224 | (26, 25, 49) | R Frontal Sup | default, default mode, mode |
| 225 | (51, 31, 5) | R Frontal Inf Tri | negative neutral, inferior frontal, videos |
| 226 | (-1, 25, 46) | L Supp Motor Area | semantic, anterior prefrontal, task |
| 227 | (-45, 34, -8) | L Frontal Inf Orb | meaning, semantic, inferior frontal |
| 228 | (47, 36, -9) | R Frontal Inf Orb | conveyed, lateral orbitofrontal, problems |
| 229 | (-9, 30, 55) | L Frontal Sup Medial | dorsomedial, dorsomedial prefrontal, dmpfc |
| 230 | (29, 33, -15) | R Frontal Inf Orb | orbitofrontal cortex, orbitofrontal, ofc |
| 231 | (-28, 33, -16) | L Frontal Inf Orb | orbitofrontal cortex, orbitofrontal, food |
| 232 | (0, 29, -10) | R Frontal Med Orb | subgenual, ventromedial prefrontal, vmpfc |
| 233 | (8, 28, 30) | R Cingulum Mid | anterior cingulate, cingulate, anterior |
| 234 | (-22, 34, 44) | L Frontal Mid | retrosplenial, experiences, success |
| 235 | (-34, 35, 34) | L Frontal Mid | dorsolateral prefrontal, dorsolateral, preparation |
| 236 | (38, 37, 29) | R Frontal Mid | dorsolateral, dorsolateral prefrontal, prefrontal |
| 237 | (-43, 37, 19) | L Frontal Mid | phonological, retrieval, word |
| 238 | (12, 36, 51) | R Frontal Sup | impulsivity, trait, regional gray |
| 239 | (45, 41, 16) | R Frontal Mid | ventrolateral, ventrolateral prefrontal, working |
| 240 | (27, 37, 40) | R Frontal Mid | gyrus medial, default network, dorsolateral prefrontal |
| 241 | (-45, 42, 5) | L Frontal Inf Tri | phonological, words, demands |
| 242 | (-3, 31, 20) | L Cingulum Ant | anterior cingulate, cingulate, anterior |
| 243 | (41, 51, -9) | R Frontal Mid Orb | progression, orbitofrontal, orbitofrontal cortex |
| 244 | (-40, 49, -8) | L Frontal Mid Orb | prefrontal, prefrontal cortex, rules |
| 245 | (-1, 37, 34) | L Frontal Sup Medial | prefrontal, retrieval, medial prefrontal |
| 246 | (41, 50, 5) | R Frontal Mid | prefrontal, nociceptive, pfc |
| 247 | (0, 43, -15) | L Rectus | ventromedial, mentalizing, ventromedial prefrontal |
| 248 | (25, 48, -14) | R Frontal Mid Orb | cortex ofc, orbitofrontal cortex, orbitofrontal |
| 249 | (-26, 49, -14) | L Frontal Mid Orb | cortex ofc, orbitofrontal, orbitofrontal cortex |
| 250 | (-8, 47, 42) | L Frontal Sup Medial | medial prefrontal, negative neutral, prefrontal |
| 251 | (27, 49, 30) | R Frontal Mid | response inhibition, signal task, stop signal |
| 252 | (-35, 51, 15) | L Frontal Mid | prefrontal, prefrontal cortex, dorsolateral |
| 253 | (-25, 48, 30) | L Frontal Mid | cingulate, networks involved, diverse |
| 254 | (11, 49, 40) | R Frontal Sup Medial | pfc, dorsolateral pfc, restricted |
| 255 | (8, 42, 18) | R Cingulum Ant | anterior cingulate, cingulate, controls hc |
| 256 | (7, 44, 1) | R Frontal Sup Medial | subsequent memory, anterior cingulate, cingulate |
| 257 | (31, 54, 18) | R Frontal Mid | noxious, prefrontal, prefrontal cortex |
| 258 | (-7, 47, -1) | L Cingulum Ant | mpfc, cingulate, cortex mpfc |
| 259 | (-6, 46, 15) | L Cingulum Ant | cingulate, salience network, medial prefrontal |
| 260 | (28, 59, -2) | R Frontal Sup Orb | anterior prefrontal, frontopolar, loop |
| 261 | (-27, 58, -1) | L Frontal Sup Orb | matrix, alteral, reasoning |
| 262 | (0, 50, 30) | L Frontal Sup Medial | medial prefrontal, posterior cingulate, default network |
| 263 | (-4, 56, -9) | L Frontal Med Orb | ventromedial, ventromedial prefrontal, mpfc |
| 264 | (-25, 58, 13) | L Frontal Sup | pictures, prefrontal, functional connectivity |
| 265 | (8, 59, -6) | R Frontal Med Orb | orbitofrontal cortex, orbitofrontal, medial |
| 266 | (22, 63, 12) | R Frontal Sup | frontopolar, age sex, comprehensive |
| 267 | (-11, 59, 25) | L Frontal Sup Medial | medial prefrontal, speaker, recollection |
| 268 | (10, 59, 25) | R Frontal Sup Medial | medial prefrontal, belief, mind tom |
| 269 | (6, 62, 9) | R Frontal Sup Medial | medial prefrontal, medial, autobiographical |
| 270 | (-9, 63, 8) | L Frontal Sup Medial | medial prefrontal, prefrontal cortex, medial |

**Supplementary Table 1: Regions of interest used in the analysis.** For each of the 270 regions, we provide MNI coordinates (second column), the matching structural brain location as derived from the AAL atlas (third column), and a few keywords indicative of the region functions retrieved from the *neurosynth* platform (fourth column).
